# Assessing the relationship of maternal short stature with coexisting forms of malnutrition among neonates, infants, and young children of Pakistan

**DOI:** 10.1002/fsn3.3945

**Published:** 2024-01-08

**Authors:** Asif Khaliq, Smita Nambiar, Yvette D. Miller, Darren Wraith

**Affiliations:** ^1^ School of Public Health and Social Work Queensland University of Technology Brisbane Queensland Australia; ^2^ School of Nutrition and Dietetics Queensland University of Technology Brisbane Queensland Australia

**Keywords:** children, coexisting form, influence, malnutrition, maternal, short stature

## Abstract

Evidence from previous studies suggests a strong association between pediatric undernutrition and maternal stature. However, there's a scarcity of evidence regarding the relationship between maternal stature and pediatric coexisting forms of malnutrition (CFM). This study examined the prevalence and trends of CFM at the individual, household, and community levels, using data from the Demographic & Health Surveys (DHS) of Pakistan. Furthermore, this study assessed the association between pediatric CFM and short maternal stature while adjusting for multiple covariates. A panel cross‐sectional analysis was conducted using data from the 2012–2013 and 2017–2018 Pakistan Demographic & Health Survey (PDHS). We included data from 6194 mother–child dyads aged 15–49 years and 0–59 months, respectively, while excluding data from pregnant mothers and dyads with incomplete anthropometric variables and anthropometric outliers. Across the two survey periods, our findings reveal a significant decline in pediatric malnutrition, including CFM, alongside a concurrent increase in maternal overweight/obesity. Three out of four households had either a malnourished mother, and/or a malnourished child, and/or both. Our study demonstrates that short maternal stature increased the odds of various forms of pediatric undernutrition by two‐to‐threefolds (*p* < .041), but we did not find an association with wasting, overweight/obesity, and nutritional paradox. This underscores the heightened vulnerability of children born to short‐stature mothers to various forms of pediatric undernutrition. Addressing the high prevalence of pediatric undernutrition among children of short‐stature mothers necessitates a comprehensive approach that considers an individual's nutritional status throughout their entire life cycle.

## Graphical abstract

Children living in most of the Asian and African countries are at highly vulnerable to various types of nutritional adversities, including undernutrition. Undernutrition during early infancy and childhood laid the foundation for intergenerational undernutrition. Maternal short stature (<145 cm) is an irreversible type of undernutrition, which is associated with various types of nutritional adversities, such as intrauterine fetal growth retardation, small‐for‐gestational age (SGA), low birth weight (LBW), and undernutrition. This study reported a high prevalence of malnutrition across Pakistan, where three out of every four households had either a malnourished child, and/or malnourished mother, and/or a malnourished mother–child dyad. The relationship of maternal short stature with CFM is not yet assessed, but this study measured significantly two‐to‐threefold higher odds of pediatric undernutrition (standalone forms and coexisting forms of undernutrition) compared with children of normal/tall heighted mothers. Tackling the pediatric undernutrition requires a life‐cycle approach, with special emphasis on all adolescent girls and expecting mothers, so that issues related to nutritional disorders that may develop in adolescent girls, expecting mothers, developing fetuses, and newborns can be curtailed early. This study investigates the link between maternal stature and pediatric coexisting forms of malnutrition (CFM) using Pakistan Demographic & Health Surveys (PDHS) data. Results indicate a decline in pediatric malnutrition and an increase in maternal overweight/obesity over time. About 75% of households of Pakistan have either a malnourished mothers and/or child and/or both. Evidence of a relationship between maternal short stature with CFM is scarce, but it showed two‐to‐threefold higher odds of pediatric undernutrition in children born to short‐stature mothers.

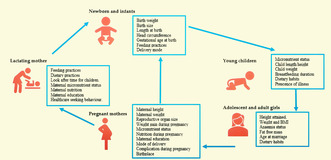

## 1 INTRODUCTION

Malnutrition is a pressing global health concern, particularly among women of reproductive age and children under the age of five (Mishu et al., 2020), and can be categorized into standalone forms of malnutrition (SFM) and coexisting forms of malnutrition (CFM). SFM indicates only one type of anthropometric disorder (deficiency or excess) in an individual, irrespective of age, whereas CFM presents as a combination of two or more indicators of malnutrition in an individual (Khaliq et al., 2021, 2022). Compared to SFM, CFM is notably associated with a fourfold higher risk of mortality in children (Garenne et al., 2019).

A child's nutritional status and growth trajectory are influenced by various maternal factors, such as maternal genetic makeup, nutrition during pregnancy, and growth pattern (from intrauterine life through adolescence and adulthood) (Azcorra & Mendez, 2018; Khatun et al., 2018). Among these factors, maternal height stands out as a strong predictor of offspring growth and nourishment (Khatun et al., 2018). Unlike maternal weight and body mass index (BMI), maternal height remains constant after maternal puberty (Khatun et al., 2019). Mothers with a height below 145 cm, either before conception or after childbirth, are classified as having “short stature” (Khatun et al., 2019; Subramanian et al., 2009). Several studies have shown a higher risk of intrauterine complications and post‐uterine growth failure in children born to mothers with short stature. The risk of growth failure and related nutritional disorders, such as intrauterine fetal growth retardation, small‐for‐gestational age (SGA), low birth weight (LBW), and undernutrition, is two to four times higher in children of short‐statured mothers (<145 cm) compared to those of normal or tall mothers (Mertens et al., 2020; Rahman et al., 2016). Thus, children of mothers with short stature are more vulnerable to experiencing growth failure and related nutritional disorders.

Worldwide, over half of children in South Asian and Sub‐Saharan African countries suffer from various types of malnutrition (Akhtar, 2016; Stephenson et al., 2000). In Pakistan, CFM affects more than two‐thirds of the population. Pakistan is the second‐largest South Asian country after India and has witnessed a stagnation of certain types of malnutrition in children over the last four decades. This stagnation may be partially attributed to the intergenerational cycle of malnutrition (National Institute of Population Studies (NIPS), P., ICF, 2019; United Nations Children Funds, 2019). The first 1000 days of life offer a critical window of opportunity to break the intergenerational cycle of malnutrition from parents to offspring (Estrada‐Gutiérrez et al., 2020). Interventions applied during this period can effectively avert various types of nutritional disorders, including CFM, thereby safeguarding children from numerous illnesses through simple and cost‐effective interventions (Estrada‐Gutiérrez et al., 2020; Jones et al., 2003).

CFM is a relatively new concept, with limited studies exploring its prevalence, trends, and determinants in children (Khaliq et al., 2022; Pradeilles et al., 2023; Sumon et al., 2023). Previous research, including our own analysis of PDHS datasets, has highlighted the socioeconomic determinants of CFM in children aged below 5 years (Khaliq et al., 2021). Several studies have reported that short maternal stature is a proxy measure for maternal malnutrition, which in turn affects the growth trajectory of the developing fetus, and of young children aged below 5 years (Khatun et al., 2019; Mertens et al., 2020; Rahman et al., 2016). However, direct evidence of an association between short maternal stature and CFM in children remains unreported to date. Therefore, this study aims to determine the prevalence and trends of various types of CFM among mother–child dyads and assess the relationship of maternal short stature with pediatric CFM among neonates, infants, and children of Pakistan.

### 1.1 Key messages

- Children living in most of the Asian and African countries are highly vulnerable to various types of nutritional adversities, including undernutrition.
- Undernutrition during early infancy and childhood can lay the foundation for intergenerational undernutrition. Maternal short stature, defined as a height below 145 cm, represents an irreversible form of undernutrition and is associated with a range of nutritional adversities, including intrauterine fetal growth retardation, small‐for‐gestational age (SGA), low birth weight (LBW), and undernutrition.
- This study reported a high prevalence of malnutrition across Pakistan, where three out of every four households had at least one member who is malnourished—a child, a mother, or a mother–child dyad. Evidence of a relationship between maternal short stature with CFM is scarce, but this study found a two‐to‐threefold higher odds of pediatric undernutrition (standalone forms and coexisting forms of undernutrition) among children born to mothers with short stature compared to those born to normal or tall mothers.
- Tackling pediatric undernutrition necessitates a comprehensive life‐cycle approach, with a particular focus on adolescent girls and expectant mothers. This approach aims to proactively address nutritional disorders that may arise during adolescence, pregnancy, fetal development, and the early stages of newborns.

## 2 STUDY METHODOLOGY

### 2.1 Data source

This study involved secondary data analysis of the PDHS (National Institute of Population Studies (NIPS), P., ICF, 2019). In Pakistan, the National Institute of Population Studies conducted four demographic health surveys in 1990–1991, 2006–2007, 2012–2013, and 2017–2018, respectively. For this research, this study utilized datasets from the last two PDHS surveys. Among all the PDHS conducted in Pakistan, the 2017–2018 PDHS was the only one that collected data across all of Pakistan. However, in other PDHS surveys conducted before 2017–2018, data were not collected from Azad Jammu and Kashmir (AJK) and Federally Administered Tribal Areas (FATA) regions due to geopolitical and security reasons. We discuss the implications of this difference in the Discussion section.

### 2.2 Study population and eligibility criteria

This study included mother–child dyads, with mothers aged 15–49 years and children aged 0–59 months. Data from mother–child dyads containing comprehensive information on maternal weight, maternal height, child's weight, and child's height were analyzed. However, data from dyads that included pregnant mothers and either infants or mothers whose height/length and weight were considered anthropometric outliers and were excluded (Appendix S1: Supplementary file 1). Anthropometric outliers are biologically implausible values that may occur owing to measurement errors, reporting errors, and data entry errors (Phan et al., 2020). The upper and lower limits for anthropometric outliers for various anthropometric indices based on z‐score values were: ±6.00 S.D. for HAZ/LAZ; ±5.00 S.D. for WHZ; and −6.00 and +5.00 S.D. for WAZ (World Health Organization, 2019).

### 2.3 Sampling strategy and sample size

The sampling frames in each PDHS were adopted from the Pakistan Bureau of Statistics (PBS) and the last census record of 2017. A multi‐stage stratified cluster systematic sampling technique was used for the selection of each household. Further details regarding the sample selection have been reported elsewhere (Khaliq et al., 2021).

The sample size of the PDHS was calculated from the list of enumeration blocks (EBs). In 2012–2013, 500 EBs were selected, while in the 2017–2018 survey, 580 EBs were selected. The total sample size for each PDHS was calculated by multiplying the number of EBs selected with a constant number of 28. Thus, the sample size calculated for the 2012–2013 survey was 14,000 and the 2017–2018 survey was 16,240. The actual dataset contained data from 11,763 eligible women in 2012–13 and 12,708 in 2017–18 (or 24,471 women in total). After excluding cases with missing/incomplete anthropometric values, anthropometric outliers, and/or pregnant mothers, 6194 mother–child dyads were available for these analyses (Appendix S1: Supplementary file 1). For evaluating the statistical power of this study, a post‐hoc power calculator for prevalence studies was used, and we observed a statistical power of >80%.

### 2.4 Measurement of nutritional status and prevalence of CFM

In this study, anthropometric data were used for calculating the nutritional status of each mother–child dyad. For assessing the nutritional status of each child, z‐scores of three common anthropometric indices: weight‐for‐age (WAZ), weight‐for‐height (WHZ), and height‐for‐age (HAZ) were calculated using WHO AnthroCal® software. Subsequently, five different types of nutritional status (normal, wasted, stunted, underweight, and overweight/obese) were identified based on the z‐score values of each anthropometric index. The presence or absence of CFM was then derived based on deviant z‐scores for any two or more of the anthropometric indices. Children presenting with one deviant Z‐score were coded as having a standalone form of malnutrition (Khaliq et al., 2021). Thus, we identified four types of standalone malnutrition (wasting, stunting, underweight, and overweight/obesity) and four types of CFM (coexisting underweight with wasting, underweight with stunting, underweight with both wasting and stunting, and stunting with overweight/obesity) in children below 5 years of age (Appendix S1: Supplementary file 2).

Maternal weight and height information was used for assessing maternal nutritional status using a multilevel assessment approach. First, the body mass index (BMI) of each mother was calculated using the BMI standard formula, and mothers were categorized as underweight, normal, and overweight/obese based on their obtained BMI values. Second, mothers were categorized as having short stature or normal stature based on their height. Mothers with a height of 145 cm or more were classified as “normal,” while mothers who were shorter than this were classified as being of short stature. Finally, analysis was performed for identifying maternal standalone forms of malnutrition and CFM (Appendix S1: Supplementary file 3).

CFM at the individual level was divided into maternal and child CFM. CFM at the household level represented the concurrent existence of pediatric CFM, or standalone forms of malnutrition, with maternal CFM, or standalone forms of malnutrition. CFM at the community level represented the proportion of households that reported CFM in either an individual or a household. CFM at the community level was coded into one of three categories: (1) *Healthy households*, where the anthropometric measurements of both mother and child were normal. (2) *Households with an affected individual*: where either the mother or child showed deviated anthropometric measurements against one or more anthropometric indices; and (3) *Households with affected mother–child dyads*: where both the mother and child showed deviated anthropometric measurements against one or more anthropometric indices. The prevalence and trends of malnutrition at the community level were assessed by aggregating the cumulative statistics obtained from the individual and household levels for each year.

### 2.5 Measurement of maternal height

Two categories of maternal height were created: *short stature* (<145 cm) and *normal stature* (≥145 cm). These categories of maternal height were adopted from previous studies conducted in different Asian countries sharing similar types of ethnic and sociodemographic characteristics (Khatun et al., 2019; Subramanian et al., 2009).

### 2.6 Study covariates

**FIGURE 1:**
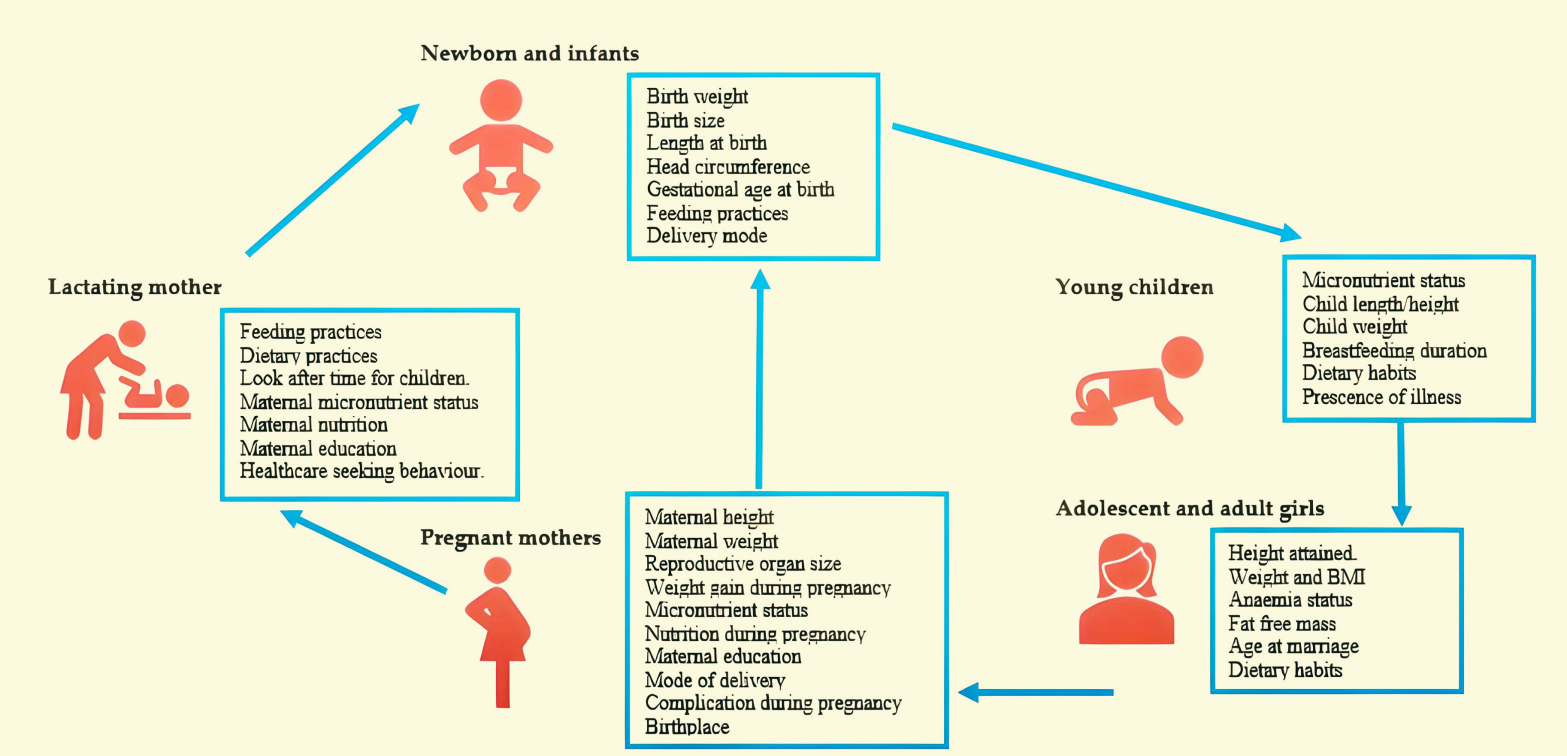
Cycle of malnutrition between the malnourished mother and offspring. Adapted from: Martorell and Zongrone (2012), Sumarmi et al. (2016), and Wali et al. (2019).

The relationship between maternal short stature with pediatric malnutrition, including CFM depends on a wide range of factors. These factors include demographic and housing conditions. To assess the relationship between pediatric CFM and short maternal status, a conceptual framework was developed to illustrate how these factors interacted. This framework was informed by the framework proposed by UNICEF (2013) (Wali et al., 2019) and other studies (Martorell & Zongrone, 2012; Sumarmi et al., 2016). In children, malnutrition has multilevel factors: immediate, underlying, and basic (Wali et al., 2019) (Figure 1).

Following this framework, we identified and categorized various covariates from the dataset and grouped them into two major domains: child factors and maternal factors.

1. Child factors: Age (five categories: 0–11 months, 12–23 months, 24–35 months, 36–47 months, and 48–59 months), gender, illness history (yes/no), birth order (primigravida/multigravida), and birth size (large, average, and small) of each child were included in analyses. Birthweight could not be considered because the birthweight of more than 70% of children was unknown. However, we included birth size, which was a proxy measure of birthweight, but the data related to birthweight was collected subjectively via maternal response/recall.
2. Maternal factors: We included maternal age (less than 20 years, between 20 and 34 years, and 35 years or more), maternal education (none, primary, and secondary/higher), maternal work status (yes/no), maternal empowerment to healthcare (yes/no), maternal body mass index (BMI) (normal, i.e., 18.5–24.9 kg/m^2^, underweight, i.e., <18.5 kg/m^2^, and overweight/obese, i.e., ≥25 kg/m^2^), delivery by C‐section (yes/no), total number of pregnancies, and whether mothers had been pregnant in the last year (yes/no) as maternal factors. Maternal empowerment is a composite variable, which was examined through a variety of questions assessing maternal ability related to healthcare seeking behavior, maternal involvement in capital decisions, such as the purchase of household stuff and property, and maternal freedom (National Institute of Population Studies (NIPS), P., ICF, 2019).

Place of residence (urban or rural), wealth index (classified as poorest, poor, middle, richer, or richest), place of birth (home delivery or hospital‐based delivery), and family size were integrated into the model as control variables. This inclusion enabled us to examine the potential influence of these variables while assessing the relationship between maternal and child factors and pediatric CFM.

### 2.7 Statistical analysis

Each PDHS dataset was analyzed separately. Initially, descriptive analysis was performed for each dataset. Following descriptive analysis, the difference in the distribution of predictor, outcome, and other covariates between two survey periods was assessed using chi‐square and independent‐sample *t*‐test (Table 1). For assessing the prevalence of malnutrition, including various forms of CFM among mother–child dyads, we calculated the prevalence estimates for each type of malnutrition, including CFM. The prevalence estimates of each form of malnutrition were converted into percentages. The difference in the prevalence of malnutrition across two survey periods was assessed by comparing the 95% confidence interval (CI) limit of one survey period with another.

**TABLE 1:** Sociodemographic and household characteristics of the study sample.

Variable	Category	2012–2013 PDHS (*n* = 2637)	2017–2018 PDHS (*n* = 3557)	Total (*n* = 6194)	*p*‐Value
Maternal factors					
BMI categorization	Normal	53.2%	45.7%	48.9%	<.001
	Underweight	13%	9.3%	10.9%	
	Overweight/Obese	33.8%	45%	40.2%	
Maternal height	Normal stature	95%	96%	95.4%	.041
	Short stature	5%	4%	4.4%	
Maternal age at time of survey	15–19 years	2.2%	2.8%	2.6%	.016
	Between 20 and 34 years	74.4%	76.5%	75.6%	
	35 years or more	23.4%	20.7%	21.8%	
Maternal education	None	53.1%	49.3%	50.9%	<.001
	Primary	16.5%	14.1%	15.1%	
	Secondary/higher	30.4%	36.5%	33.9%	
Maternal working status	Yes	21.4%	11.6%	15.8%	<.001
	No	78.6%	88.4%	84.2%	
Maternal empowerment	Yes	46.7%	87.2%	67.8%	<.001
	No	53.3%	12.8%	32.2%	
Total children delivered	–	4.0 ± 2.4	3.7 ± 2.2	3.8 ± 2.3	<.001
Birth in last year	No	58%	56.5%	57.1%	.246
	Yes	42%	43.5%	42.9%	
Delivery by C‐section	No	86.8%	81%	83.4%	<.001
	Yes	13.2%	19%	16.6%	
Child factors					
Child age	0–11 months	19%	22%	20.7%	.005
	12–23 months	17.3%	19.1%	18.4%	
	24–35 months	21%	19.3%	20%	
	36–47 months	21.2%	19.6%	20.2%	
	48–59 months	21.5%	20%	20.7%	
Sex of child	Male	51%	51.3%	51.2%	.831
	Female	49%	48.7%	48.8%	
History of illness	Yes	53.7%	55.3%	55.4%	.888
	No	46.3%	44.7%	44.6%	
Birth size	Average	73.6%	75.7%	74.8%	.145
	Large	8.2%	7.6%	7.9%	
	Small	18.3%	16.6%	17.3%	
Household and community factors					
Wealth index	Poorest	19.9% 20.6%	24.7%	20.6%	<.001
	Poorer	18.8%	20.6%	22.7%	
	Middle	20.9%	19.3%	19.1%	
	Richer	19.8%	17.6%	19%	
	Richest		17.8%	18.6%	
Place of delivery	Home	46.2%	33.3%	38.8%	<.001
	Hospital	53.8%	66.7%	61.2%	
Family size	–	9.4 ± 5.4	9.5 ± 4.9	9.4 ± 5.1	.843
Type of place of residence	Urban	43.2%	45.8%	44.7%	.037
	Rural	56.8%	54.2%	55.3%	

The relationship between maternal height and various forms of malnutrition were initially assessed relative to a normal healthy child (rather than within) using bivariate and multivariable logistic regression (Table 3). Subsequently, subgroup analyses were performed, where the relationship of each type of CFM was compared with standalone forms of malnutrition (underweight and stunting) using bivariate (Appendix S1: Supplementary file 4) and multivariable logistic regression (Table 4). The reference category for various types of coexisting forms of undernutrition (underweight with wasting, underweight with stunting, and underweight with both wasting and stunting) was underweight, while for the coexistence of stunting with overweight/obesity, the reference category was stunting. Before performing multivariate analysis, the collinearity of each covariate was assessed, and variables with high VIF values (>10) were removed from the model. In the multivariable regression model, all covariates were entered together, and a backward elimination method was applied to eliminate non‐significant variables (using a *p*‐value threshold of .10).

### 2.8 Ethical clearance

The data used in this study were obtained from the DHS data repository after formal registration and approval. Additionally, the protocol of this study received approval from the University Human Research Ethics Committee (UHREC) of the Queensland University of Technology (QUT), Brisbane, Australia (Approval number 2000000177).

## 3 RESULTS

This study analyzed data from 6194 eligible mother–child dyads from 2012 to 2013 and 2017 to 2018 PDHS datasets. Table 1 provides a comparison of the two survey periods for a range of sociodemographic and household characteristics. Except for birth in the last year, child sex, history of illness, birth size, and family size, all other differences between the two survey periods were statistically significant (Table 1).

### 3.1 Prevalence and trends of CFM at individual, household, and community levels

Table 2 presents the prevalence of SFM and CFM among mothers and children at the individual level.

**TABLE 2:** Nutritional profile of women of reproductive age and children under 5 years of age.

Variables and their categories	PDHS 2012–13 (*n* = 2637) (95% CI)	PDHS 2017–18 (*n* = 3557) (95% CI)
Maternal (women of reproductive age) nutritional profile		
Normal	51% (49.1–52.9%)*	43.6% (41.9%–45.2%)*
Malnourished	49% (48.1%–50.9%)*	56.4% (54.8%–58.1%)*
Standalone forms of malnutrition	46.2% (42.5%–49.8%)*	54.3% (51.3%–57.5%)*
Underweight	11.9% (10.6%–13.2%)*	9.1% (8.1%–10.1%)*
Overweight/obesity	32% (30.2%–33.8%)*	43.4% (41.7%–44.9%)*
Short stature	2.2% (1.7%–2.8%)	2% (1.5%–2.5%)
Coexisting forms of malnutrition	2.8% (1.9%–4.2%)	2.0% (1.3%–2.6%)
Coexistence of underweight with short stature	1.1% (0.7%–1.5%)	0.3% (0.1%–0.5%)
Coexistence of overweight/obesity with short stature	1.7% (1.2%–2.2%)	1.7% (1.2%–2.1%)
Children (under 5 years of age) nutritional profile		
Normal	45.9% (44–47.8%)*	57.7% (56%–59.2%)*
Malnourished	54.1% (52.2%–56%)*	42.3% (40.8%–44%)*
Standalone forms of malnutrition	23.4% (20.3%–26.5%)	21.6% (19.1%–24.1%)
Wasting	3.2% (2.5%–3.9%)	2.8% (2.1%–3.2%)
Stunting	17% (15.6%–18.5%)	16.2% (15.2%–17.6%)
Underweight	1.4% (1.0%–1.9%)	0.8% (0.5%–1.1%)
Overweight/obesity	1.7% (1.2%–2.2%)	1.7% (1.3%–2.2%)
Coexisting forms of malnutrition	30.7% (26.9–34.6%)*	20.7% (18%–23.3%)*
Coexistence of underweight with wasting	3.1% (2.4%–3.8%)	3% (2.4%–3.5%)
Coexistence of underweight with stunting	17.9% (16.4%–19.3%)*	13.6% (12.4%–14.7%)*
Coexistence of underweight with wasting & stunting both	4.4% (3.6%–5.2%)*	2.6% (2.1%–3.2%)*
Coexistence of stunting with overweight/obesity	5.4% (4.5%–6.3%)*	1.5% (1.1%–1.9%)*

Abbreviation: CI, confidence interval.

* *p*‐value ≤.05, that is, significance difference in prevalence of malnutrition across two survey periods.

The prevalence of maternal malnutrition significantly increased from 49% (48.1%–50.9%) in 2012–2013 to 56.4% (54.8%–58.1%) in 2017–18. This was mainly attributed to maternal overweight and obesity, which increased by over 10% between the two survey periods. The prevalence of underweight declined from 11.9% (10.6%–13.2%) in 2012–2013 to under 9.1% (8.1%–10.1%) in 2017–18. The prevalence of CFM among mothers was less than 5% in both survey periods.

The prevalence of child malnutrition decreased from 54.1% (52.2%–56%) in 2012–2013 to 42.3% (40.8%–44%) in 2017–2018. The prevalence of CFM in children decreased from 30.7% (26.9%–34.6%) to 20.7% (18%–23.3%) across the survey periods. Coexisting underweight and stunting were the most prevalent type of CFM in both time periods. The prevalence of various type of CFM had declined in 2017–2018 compared to 2012–2013, except for coexisting underweight with wasting (which remained stable).

At the household level, the prevalence of malnutrition decreased from 26.1% in 2012–2013 to 22.5% in 2017–2018. Maternal CFM was present in less than 1% of households for both time periods, while child CFM was present in 14.1% of households in 2012–13 and decreased to 10.1% in 2017–18. The presence of CFM in the mother–child dyad was 1.1% in 2012–13 and decreased to 0.5% in 2017–18.

**FIGURE 2:**
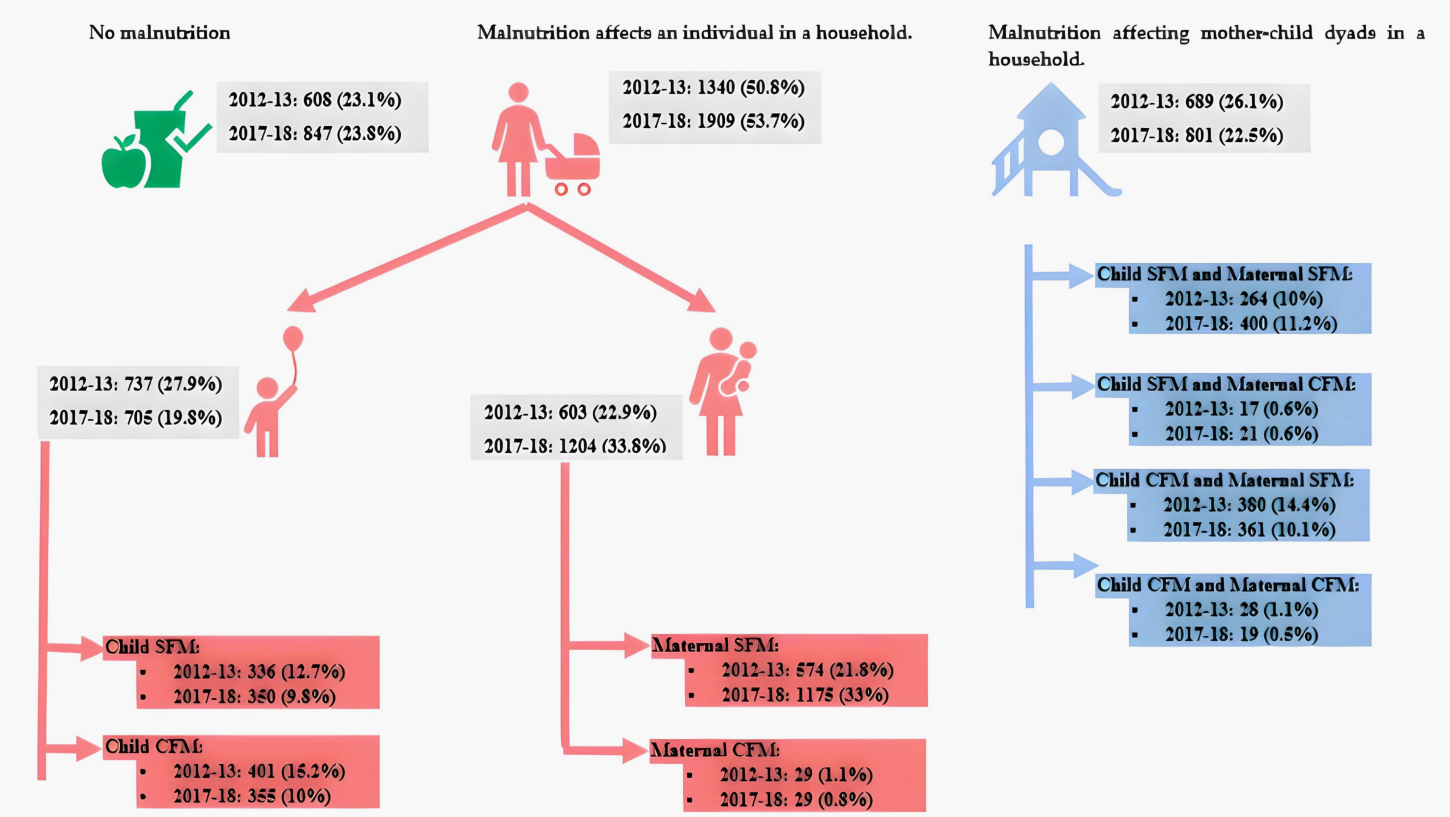
Maternal and child nutritional status at the household level and the community level.

At the community level, we observed a healthy nutritional status in less than a quarter of mother and/or child and/or mother–child dyads across the two survey periods (Figure 2).

### 3.2 Determinants of coexisting forms of undernutrition

The short stature of mothers was significantly associated with various forms of undernutrition, including coexisting forms of undernutrition. We found at least a twofold higher odds of stunting, underweight, and all forms of coexisting forms of undernutrition, such as coexisting underweight with wasting, underweight with stunting, and underweight with both wasting and stunting, in children of mothers with short stature compared to mothers of normal stature (Table 3). However, we did not observe a relationship between maternal height and various coexisting forms of undernutrition when compared with an underweight child as the reference (as the standalone form of malnutrition) (Table 4).

**TABLE 3:** Determinants of standalone and coexisting forms of undernutrition in children below 5 year of age using PDHS data of 2012–13 and 2017–18 (Reference is a normal child).

Variable	Categories	Standalone forms of malnutrition^a^				Coexisting forms of malnutrition^b^			
		Wasting^c^	Stunting^c^	Underweight^c^	Overweight/obesity^c^	Coexistence of underweight with wasting^c^	Coexistence of underweight with stunting^c^	Coexistence of underweight with wasting and stunting^c^	Coexistence of stunting with overweight/obesity^c^
Maternal factors									
Maternal height	Normal/tall stature	Ref	Ref	Ref	Ref	Ref	Ref	Ref	Ref
	Short stature	1.90 (0.96–3.76)	2.67 (1.11–6.44) *	1.74 (1.22–2.47) *	0.98 (0.30–3.21)	2.69 (1.51–4.80) *	2.01 (1.43–2.83) *	1.90 (1.02–3.51) *	1.54 (0.76–3.14)
Maternal age	15–19 years	–	–	–	–	–	–	–	–
	20–34 years								
	35‐year or more								
Maternal education	No education	Ref	Ref	Ref	Ref	Ref	Ref	Ref	Ref
	Primary	0.58 (0.35–0.95)*	1.36 (0.69–2.67)	0.86 (0.69–1.07)	0.77 (0.41–1.44)	0.69 (0.43–1.12)	0.78 (0.62–0.98)*	0.56 (0.35–0.91) *	0.58 (0.34–0.97)*
	Secondary or higher	0.64 (0.41–1.00)	0.74 (0.35–1.58)	0.81 (0.66–1.01)	0.76 (0.43–1.33)	0.88 (0.57–1.36)	0.67 (0.54–0.84)*	0.53 (0.34–0.84)*	0.71 (0.46–1.11)
Maternal working status	No	–	–	–	–	–	–	–	–
	Yes								
Maternal empowerment for healthcare	No	Ref	Ref	Ref	Ref	Ref	Ref	Ref	Ref
	Yes	0.69 (0.50–0.96)*	0.64 (0.38–1.09)	0.83 (0.71–0.98)*	0.65 (0.43–1.00)	0.82 (0.58–1.14)	0.73 (0.62–0.86)*	1.90 (1.02–3.51)*	0.42 (0.31–0.57)*
Total children delivered		0.91 (0.55–1.51)	0.47 (0.22–1.00)	0.96 (0.78–1.18)	1.19 (0.69–2.07)	0.69 (0.44–1.08)	0.77 (0.63–0.96)*	0.61 (0.40–0.92)*	1.79 (1.18–2.71)*
Birth in last year	No	–	–	–	–	–	–	–	–
	Yes								
Delivery by C‐section	No	Ref	Ref	Ref	Ref	Ref	Ref	Ref	Ref
	Yes	0.48 (0.28–0.81)*	1.15 (0.58–2.28)	0.82 (0.66–1.01)	1.21 (0.72–2.02)	0.50 (0.29–0.86)	0.67 (0.53–0.85)*	0.74 (0.45–1.23)	0.94 (0.61–1.47)
Child factors									
Age	0–11 months	Ref	Ref	Ref	Ref	Ref	Ref	Ref	Ref
	12–23 months	0.71 (0.47–1.06)	0.23 (0.09–0.61) *	3.03 (2.26–4.05)*	0.54 (0.31–0.97)*	0.77 (0.49–1.22)	1.61 (1.24–2.08)*	1.18 (0.79–1.77)	0.38 (0.21–0.69)*
	24–35 months	0.50 (0.31–0.82)*	0.49 (0.21–1.14)	5.15 (3.85–6.90)*	0.43 (0.22–0.84)*	1.05 (0.66–1.69)	2.63 (2.02–3.41)*	0.85 (0.52–1.39)	1.11 (0.70–1.76)
	36–47 mo	0.51 (0.29–0.86)*	0.57 (0.22–1.42)	4.78 (3.49–6.54)*	0.28 (0.12–0.64)*	0.83 (0.48–1.45)	2.73 (2.05–3.63)*	0.83 (0.47–1.46)	0.80 (0.47–1.35)
	48–59 mo	0.14 (0.06–0.31)*	0.92 (0.38–2.19)	2.78 (1.98–3.91)*	0.50 (0.23–1.05	0.73 (0.41–1.32)	1.77 (1.29–2.42)*	0.88 (0.49–1.57)	0.48 (0.26–0.87)*
Sex	Male	–	–	–	–	–	–	–	–
	Female								
Presence of illnesses	No	–	–	–	–	–	–	–	–
	Yes								
Birth size	Average	Ref	Ref	Ref	Ref	Ref	Ref	Ref	Ref
	Large	1.08 (0.62–1.87)	0.85 (0.31–2.41)	0.95 (0.73–1.24)	1.38 (0.74–2.59)	0.39 (0.17–0.91)*	0.72 (0.53–0.98)*	0.47 (0.21–1.04)	1.05 (0.61–1.81)
	Small	0.93 (0.62–1.41)	1.60 (0.91–2.81)	0.96 (0.78–1.17)	0.71 (0.38–1.28)	1.13 (0.78–1.65)	1.24 (1.02–1.51)*	1.45 (1.03–2.04)*	0.90 (0.59–1.36)
Community and household factors									
Socioeconomic status	Poorest	Ref	Ref	Ref	Ref	Ref	Ref	Ref	Ref
	Poorer	0.95 (0.61–1.51)	0.76 (0.38–1.52)	0.77 (0.62–0.96)*	0.88 (0.45–1.71)	1.07 (0.70–1.65)	0.60 (0.48–0.75)*	0.75 (0.51–1.10)	0.70 (0.45–1.07)
	Middle	1.05 (0.64–1.73)	0.59 (0.26–1.33)	0.68 (0.53–0.87)*	0.84 (0.41–1.73)	0.65 (0.39–1.09)	0.56 (0.43–0.72)*	0.39 (0.24–0.64)*	0.51 (0.31–0.86)*
	Richer	0.72 (0.39–1.31)	0.31 (0.11–0.85)*	0.51 (0.38–0.67)*	0.93 (0.43–2.00)	0.50 (0.28–0.89)*	0.59 (0.45–0.78)*	0.37 (0.21–0.63)*	0.44 (0.25–0.78)*
	Richest	0.91 (0.48–1.73)	0.46 (0.16–1.33)	0.47 (0.35–0.65)*	0.95 (0.41–2.21)	0.25 (0.12–0.52)*	0.43 (0.31–0.60)*	0.17 (0.08–0.36)*	0.61 (0.33–1.12)
Place of birth	Home	–	–	–	–	–	–	–	–
	Hospital								
Family size		1.01 (0.97–1.04)	0.98 (0.93–1.04)	1.02 (1.01–1.04)*	1.01 (0.97–1.05)	1.03 (1.00–1.06)*	1.01 (0.99–1.02)	1.03 (1.01–1.06)*	1.01 (0.97–1.03)
Type of place of residence	Rural	Ref	Ref	Ref	Ref	Ref	Ref	Ref	Ref
	Urban	0.93 (0.65–1.34)	1.05 (0.58–1.89)	1.14 (0.96–1.35)	1.29 (0.81–2.06)	1.64 (1.17–2.32)*	1.00 (0.83–1.19)	1.71 (1.22–2.38)*	1.44 (1.01–2.05)*

*Note*: Ref = Reference category of various covariates. The Reference category for maternal height was normal/tall stature, the reference category of maternal age was 15–19 years, the reference category of maternal education was no education, the reference category of maternal working status was no, the reference category of maternal empowerment was no, the reference category of birth in last year was no, the reference category of delivery by C‐section was no, the reference category of child age was 0–11.9 months, the reference category of child sex was male, the reference category of presence of illness was no, the reference category of child birth size was average, the reference category of survey year was 2012, the reference category of socioeconomic status was poorest, the reference category of place of delivery was home, and the reference category of place of residence was rural.

* *p*‐value ≤.05. That is, significant association with the study outcome.

^a^ The reference category for assessing the determinants of standalone forms of undernutrition was a normal healthy child.

^b^ The reference category for assessing the determinants of coexisting forms of undernutrition was a normal healthy child.

^c^ Adjusted for maternal height, maternal education, maternal empowerment, delivery by C‐section, child's age in months, birth size, wealth index, family size, and place of residence.

**TABLE 4:** Assessing the adjusted Odds of various forms of Coexisting forms of malnutrition.

Variable	Categories	Coexistence of underweight with wasting^a^		Coexistence of underweight with stunting^a^		Coexistence of underweight with wasting and stunting both^a^		Coexistence of stunting with overweight/obesity^b^	
		Adjusted odds^c^ (95% CI)	*p*‐Value	Adjusted odds^d^ (95% CI)	*p*‐Value	Adjusted odds^e^ (95% CI)	*p*‐Value	Adjusted odds^f^ (95% CI)	*p*‐Value
Maternal factors									
Maternal height	Normal/tall stature	Ref	.617	Ref	.456	Ref	.573	Ref	.916
	Short stature	0.76 (0.26–2.21)		0.69 (0.28–1.73)		0.73 (0.25–2.11)		1.04 (0.48–2.21)	
Maternal age	15–19 years	–		–		–		Ref	.032
	20–34 years							0.27 (0.11–0.71)*	
	35 year or more							0.33 (0.12–0.90)*	
Maternal education	No education							–	
	Primary								
	Secondary or higher								
Maternal working status	No	Ref	.026						
	Yes	0.41 (0.19–0.89)*							
Maternal empowerment for healthcare	No	–							
	Yes								
Total children delivered									
Birth in last year	No								
	Yes								
Delivery by C‐section	No	Ref	.030						
	Yes	0.37 (0.16–0.90)*							
Child factors									
Child age	0–11 months	Ref	.024	Ref	<.001	Ref	.006	Ref	<.001
	12–23 months	4.11 (1.40–12.06)*		7.54 (2.85–19.93)*		5.31 (1.91–14.73)*		0.15 (0.07–0.28)*	
	24–35 months	2.86 (1.15–7.09)*		6.57 (2.93–14.71)*		1.91 (0.77–4.68)		0.31 (0.19–0.51)*	
	36–47 months	2.04 (0.81–5.14)		6.71 (2.99–15.02)*		1.46 (0.58–3.65)		0.27 (0.16–0.46)*	
	48–59 months	1.34 (0.60–3.01)		3.04 (1.56–5.94)*		1.13 (0.52–2.46)		0.36 (0.21–0.62)*	
Child Sex	Male	–		–		–		–	
	Female								
Child Presence of illnesses	No								
	Yes								
Childbirth size	Average								
	Large								
	Small								
Periodic factor									
Year	2012–2013	Ref	.049	–		–		Ref	<.001
	2017–2018	1.82 (1.00–3.34)*						0.28 (0.20–0.41)*	
Household and community factors									
Socioeconomic status	Poorest	–		–		–		–	
	Poorer								
	Middle								
	Richer								
	Richest								
Family size						1.07 (1.00–1.15)*	.029		
Place of birth	Home					–			
	Hospital								
Type of place of residence	Rural								
	Urban								

*Note*: Ref = Reference category of various covariates. The Reference category for maternal height was normal/tall stature, the reference category of maternal age was 15–19 years, the reference category of maternal education was no education, the reference category of maternal working status was no, the reference category of maternal empowerment was no, the reference category of birth in last year was no, the reference category of delivery by C‐section was no, the reference category of child age was 0–11.9 months, the reference category of child sex was male, the reference category of presence of illness was no, the reference category of child birth size was average, the reference category of survey year was 2012, the reference category of socioeconomic status was poorest, the reference category of place of delivery was home, and the reference category of place of residence was rural.

Abbreviation: CI, Confidence interval.

* *p*‐value ≤.05. That is, significant association with the study outcome.

^a^ The reference category for assessing the determinants of the coexistence of underweight with wasting, coexistence of underweight with stunting, and coexistence of underweight with wasting and stunting both was stunting.

^b^ The reference category for assessing the determinants of the coexistence of stunting with overweight/obesity was stunting.

^c^ The Model assessed the determinants of the coexistence of underweight with wasting with maternal height as a predictor. A Maternal height >145 cm was used as the reference. The Covariates adjusted were the year of the survey, child age, maternal work status, and type of delivery.

^d^ Model assessed the determinants of the coexistence of underweight with stunting with maternal height as a predictor. A Maternal height >145 cm was used as the reference. Covariates adjusted were child age.

^e^ Model assessed the determinants of coexistence of underweight with wasting and stunting both with maternal height as a predictor. A Maternal height >145 cm was used as the reference. The Covariates adjusted were child's age and family size.

^f^ Model assessed the determinants of the coexistence of stunting with overweight/obesity with maternal height as a predictor. A Maternal height >145 cm was used as the reference. The Covariates adjusted were child age, maternal age, and the year of the survey.

A higher prevalence of coexisting underweight with wasting was reported in the 2017–2018 survey, compared to the former 2012–2013 survey (Table 4). Maternal employment (0.41, 95% CI: 0.19–0.89) and maternal cesarean section (C‐section) (0.37, 95% CI: 0.16–0.90) were associated with significantly reduced odds of coexisting underweight with wasting compared with children of unemployed mothers and of normal vaginal delivery, while an increase in family size significantly increased the odds of coexisting underweight with both wasting and stunting by 1.07 (1.00–1.15) after adjusting for other covariates.

### 3.3 Determinants of coexistence of stunting with overweight/obesity

Maternal short stature was not associated with coexisting stunting and overweight/obesity when compared to the well‐nourished child (1.54, 95% CI: 0.76–3.14) (Table 3), and with the stunted child (1.04, 95% CI: 0.48–2.21) (Table 4). Between the two survey years, the odds of coexistence of stunting with overweight/obesity decreased significantly to 0.28 (0.20–0.41) in 2017–2018. Moreover, an increase in maternal age as well as child age significantly reduced the odds of coexistence of stunting with overweight/obesity compared to a stunted child (Table 4).

## 4 DISCUSSION

To the best of our knowledge, this is the first study to comprehensively assess the prevalence and trends of maternal and child nutritional status at individual, household, and community levels in Pakistan. We also thoroughly examined the interaction of maternal short stature with various forms of pediatric malnutrition, including various types of CFM. Previous studies conducted in Pakistan and other parts of the world have primarily focused on examining the relationship of maternal short stature with child obesity (Winichagoon, 2015; Wojcicki, 2014; Zemene et al., 2023) and only at a household or community level.

Across two survey periods, this study reported malnutrition in every three out of every four mother–child dyads of Pakistan. These findings align with global statistics and the broader literature, highlighting the high vulnerability of children under the age of five and women of reproductive age to various forms of nutritional adversities, including CFM, across Pakistan (Christian et al., 2020; Swaminathan et al., 2019).

Altogether, this study reported five different types of nutritional disorders in women of reproductive age: underweight, overweight/obesity, short stature, coexisting underweight with short stature, and overweight/obesity with short stature. Among the various forms of maternal nutritional issues, the highest prevalence was for maternal overweight/obesity over the two survey periods (i.e., 33.8% in 2012–2013 and 45% in 2017–2018), and short stature was observed in less than 5% of mothers. Despite the low prevalence of maternal short stature, this was found to be a strong predictor of child growth. We found a two‐to‐threefold higher odds of various forms of pediatric undernutrition (standalone forms and coexisting forms) in children born to short‐stature mothers compared to children of normal‐stature mothers, except for child wasting. Studies conducted in Bangladesh, Ethiopia, and India have also reported an approximately twofold higher risk of pediatric undernutrition in children of short‐stature mothers (Amaha & Woldeamanuel, 2021; Mishu et al., 2020; Porwal et al., 2021). Several studies have also consistently highlighted a strong association between maternal short stature and adverse birth outcomes, such as LBW, SGA, and various forms of childhood undernutrition, including stunting, wasting, and underweight (Child Health Epidemiology Reference Group Small‐for‐Gestational‐Age/Preterm Birth Working, G, 2015; Khan, 2019; Khatun et al., 2019). Conversely, our study did not show a significant relationship between maternal short stature with the coexistence of overweight/obesity with stunting and overweight/obesity. Several other studies have also found no association between short maternal stature and pediatric overweight/obesity (Félix‐Beltrán et al., 2021; Wilson et al., 2014).

The prevalence and risk of various types of nutritional issues in children depend on their age. We found that children aged between 12 and 23 months were most vulnerable to various types of undernutrition, including CFM. This period, known as the “first 1000 days of life,” is essential for child growth and nourishment (Ahmad et al., 2018). Nutritional problems not corrected during this period may lead to irreversible consequences (de Onis & Branca, 2016). Similarly, this study also showed 4‐to‐7.5‐fold higher odds of various forms of coexisting forms of undernutrition (coexisting underweight with wasting, underweight with stunting, and underweight with both wasting and stunting) in children aged between 12 and 23 months. These findings are aligned with the findings of a study conducted in Vietnam, which reported a twofold higher odds of undernutrition in children aged between 12 and 23 months (Beal et al., 2019). The nutritional deficiencies during early childhood may be averted by implementing various strategies, such as contraceptive utilization, promoting antenatal care, and implementing a referral system (Elmusharaf et al., 2015; Khan, 2019; Vir, 2016). Moreover, advocating for appropriate infant feeding practices via Exclusive Breastfeeding (EBF), complementary feeding, micronutrient supplementation, and timely hydration, particularly during the first 1000 days of life, can effectively protect both mother and child from various types of nutritional adversities (Chai et al., 2022; Jones et al., 2003).

Over the two survey periods, our study reported a significant decrease in pediatric malnutrition with a simultaneous increase in maternal malnutrition. The significant increase in maternal malnutrition was attributed to a rapid proliferation of maternal overweight/obesity cases in the 2017–2018 survey, compared to the 2012–2013 survey. The existing variables in our study datasets were insufficient for explaining reasons pertaining to these trends in maternal and children nutritional status, but we speculate that it may be due to a significant improvement in socioeconomic parameters across the two survey periods (National Institute of Population Studies (NIPS), P., ICF, 2019). The findings of our previous paper also supported a nutritional profile improvement associated with improved socioeconomic parameters (Khaliq et al., 2021). Moreover, the escalating trend in maternal overweight/obesity might be due to a significant influx of urban dwellings during the 2017–2018 survey period and links between rapid urbanization and increased poor dietary practices and physical inactivity (Machado‐Rodrigues et al., 2014). Furthermore, some studies have reported that adult overnutrition is a consequence of pediatric undernutrition (Caballero, 2006; Ferreira et al., 2008). A review by Caballero indicated the presence of adiposity, insulin resistance, and pre‐pubertal growth in adults who had any type of undernutrition in their childhood, including LBW and IUGR (Caballero, 2006). Besides adulthood obesity, pediatric undernutrition may predispose to various other types of adult metabolic disorders, such as cardiovascular diseases, diabetes, and dyslipidemia (Emokpae & Odungide, 2020). The high prevalence of pediatric undernutrition and maternal overnutrition also exhibits nutrition transition, which can only be averted through intersectoral collaboration (Jones et al., 2003). Interventions related to maternal care before and during pregnancy and after childbirth, such as maternal immunization, maternal micronutrient supplementation, adequate maternal caloric intake, and providing a supporting environment for maternal breastfeeding, can arrest intergenerational transmission of malnutrition from mothers to offspring (Mishu et al., 2020).

In summary, this study underscores the critical relationship of maternal short stature with various forms of pediatric nutritional disorders. Maternal short stature was a significant determinant of pediatric undernutrition, including various forms of coexisting forms of undernutrition, in Pakistan. However, maternal short stature has a complex relationship with pediatric nutritional status because it serves as both a risk factor and a consequence of pediatric malnutrition.

### 4.1 Study strengths and limitations

To the best of our knowledge, this is the first study to measure the relationship of maternal short stature with pediatric CFM. The sample selected in this study has national data coverage, and the sample calculated for each PDHS was based on estimates obtained from the PBS to represent the population (Khaliq et al., 2021). Despite the national coverage and representative sample size of each PDHS, certain limitations weaken the internal validity of this study: (1) The cross‐sectional study design of the PDHS means that causation between maternal and pediatric malnutrition cannot be established; (2) The 2012–2013 PDHS survey did not collect data from the AJK and FATA regions due to military restrictions and security reasons. The findings of this study cannot represent the exact nutritional picture for the whole of Pakistan. Owing to this, program managers and policymakers need to be careful in devising nationwide prevention and control strategies for various forms of malnutrition, including CFM, based on this evidence. (3) Variables such as birthweight, birth size, and maternal health interventions (prenatal, antenatal, and postnatal care) were not included because data were missing for over 50% of cases. These variables can determine immediate gestational outcomes, such as birthweight and birth size. Due to the exclusion of these variables, this study did not assess the effect of various interventions, such as antenatal consultations, iron and folate supplementation, and maternal general health management, nor pregnancy outcomes such as birthweight, birth size, and delivery method. (4) The nutritional status of both mother–child dyads was determined using anthropometry only. The PDHS was not designed to collect a comprehensive range of nutrition assessment measures. These include biochemical tests to assess nutrient deficiencies or excesses, physical examinations, and dietary investigations to assess nutritional intake. Maternal diet during pregnancy and after childbirth, maternal micronutrient status, and maternal physical health, such as the presence of comorbidities, are of particular importance for assessing the growth and nourishment trajectory of children (Ahmad et al., 2018). Thus, there is a need to investigate relationships between infant feeding, maternal micronutrient status, health indicators and pediatric health and nourishment. (5) The anthropometric data used in this study contained some measurement and recording errors, identified by implausible values and outliers. Incomplete anthropometric values were also evident due to caregiver refusal, child absence, and child irritability. Better training in anthropometry, equipment calibration, and adopting the routine practice of taking two or more readings for each anthropometric measures can help to identify measurement and recording errors. Data of over 15% of mother–child dyads comprising of incomplete anthropometry (7.1%) and anthropometric outliers (8.8%) were excluded in this study. Our study findings exclusively focus on those mother–child dyads with valid and complete anthropometry, potentially representing either a skewed or biased picture of the population under investigation. Hence, it is essential to acknowledge these limitations while interpreting the study findings.

## 5 CONCLUSION

CFM is a public health concern, which can affect an individual, household, and a community. CFM affects more than two‐thirds of the community in Pakistan. Despite a significant reduction in pediatric malnutrition, approximately half of children still suffered from malnutrition, and there was a rapid proliferation of maternal overweight/obesity from 2012 to 2018. Children of short‐stature mothers have a high risk of various forms of malnutrition, except for wasting, overweight/obesity, and nutritional paradox. There is a need to further explore the relationship between maternal health and infant feeding and pediatric undernutrition, including coexisting forms of undernutrition, to identify the best‐value strategies for arresting the intergenerational transmission of various forms of malnutrition and CFM in children.

## AUTHOR CONTRIBUTIONS

**Asif Khaliq:** Conceptualization (lead); data curation (lead); formal analysis (lead); investigation (lead); methodology (lead); project administration (equal); visualization (lead); writing – original draft (lead). **Yvette Miller:** Conceptualization (equal); investigation (equal); methodology (equal); supervision (supporting); writing – review and editing (equal). **Smita Nambiar:** Conceptualization (supporting); methodology (equal); project administration (supporting); supervision (equal); writing – review and editing (equal). **Darren Wraith:** Conceptualization (equal); formal analysis (lead); investigation (equal); methodology (supporting); project administration (lead); supervision (lead); writing – original draft (equal); writing – review and editing (lead).

## FUNDING INFORMATION

This research received no specific grant from any funding agency for research publication and dissemination purposes.

## CONFLICT OF INTEREST STATEMENT

The authors declare no conflicts of interest.

## ETHICS STATEMENT

The project of this study was approved by the Queensland University of Technology Human Research Ethics Committee (UHREC). The approval number of this project was 2,000,000,177.

## PATIENT CONSENT STATEMENT

In this study, the research team received data from the DHS repository, which was de‐identified, that is, it does not contain information in which a participant can either be identified or traced by any means. Due to this reason, this study does not involve a statement for informed consent.

## Supporting information


Appendix S1

## Data Availability

The data that support the findings of this study are available in the supplementary material of this article.
