# Supplementary material for: Assessing the relationship of maternal short stature with coexisting forms of malnutrition among neonates, infants, and young children of Pakistan
Source: Food Sci Nutr. 2024 Jan 8;12(4):2634–49. doi: 10.1002/fsn3.3945 (PMC11016414; doi:10.1002/fsn3.3945)
Supplement: Supplementary file 1 — Appendix S1 [file FSN3-12-2634-s001.docx]

**Supplementary file 1**

**S-1: Mother-child dyads selected for the analysis.**

Participant interviewed in 2012-13 PDHS and 2017-18 PDHS = 24,471

Number of women interviewed in2017-18 PDHS = 12,708

Number of women interviewed in 2012-2013 = 11,763

**Exclude**: (n= 7,806)

WRA w/o children < 5 year = 7,806

**Exclude**: (n= 8,209)

WRA w/o children < 5 year = 8,209

Children under 5 years = 4,499

Children under 5 years = 3,957

**Exclude**: (n= 1,320)

Pregnant mothers = 522

Incomplete anthro = 280

Anthropometric outliers = 518

**Exclude**: (n= 942)

Pregnant mothers = 568

Incomplete anthro = 241

Anthropometric outliers = 133

Mother & Child with complete anthropometry = 2,637

Mother & Child with complete anthropometry = 3,557

Data analysed = 6,194

PDHS = Pakistan Demographic and Health Surveys, WRA = Women of Reproductive Age, w/o = Without, Incomplete Anthro. = The dataset has missing information about the child age or sex, or weight, or length/height, or measurement method (standing/recumbent), outliers= The z-scores calculated for HAZ/LAZ exceeds over ±6.00 S.D., WHZ exceeds over ±5.00 S.D., and of WAZ must exceed -6.00 and +5.00 S.D., respectively

**Supplementary file 2**

**S-2: Clinical interpretation of various types of nutritional status in children using z-score.**

| **WAZ** | **WHZ** | **HAZ** | **Clinical interpretation** |
| --- | --- | --- | --- |
| Between -1.99 to +1.99 | Between -1.99 to +1.99 | Between -1.99 to +1.99 | Normal |
| Between -1.99 to +1.99 | Between -1.99 to +1.99 | Less than or equal to -2.00 | Stunting |
| Less than or equal to -2.00 | Between -1.99 to +1.99 | Between -1.99 to +1.99 | Underweight |
| Between -1.99 to +1.99 | Less than or equal to -2.00 | Between -1.99 to +1.99 | Wasting |
| Between -1.99 to +1.99 | Over or equal to +2.00 | Between -1.99 to +1.99 | Overweight/obesity |
| Less than or equal to -2.00 | Less than or equal to -2.00 | Between -1.99 to +1.99 | Coexistence of underweight with wasting |
| Less than or equal to -2.00 | Between -1.99 to +1.99 | Less than or equal to -2.00 | Coexistence of underweight with stunting |
| Less than or equal to -2.00 | Less than or equal to -2.00 | Less than or equal to -2.00 | Coexistence of underweight with wasting and stunting |
| Between -1.99 to +1.99 | Over or equal to +2.00 | Less than or equal to -2.00 | Coexistence of stunting with overweight/obesity |

**Supplementary file 3**

**S-3.1: Body Mass Index (BMI) Categorization**

1. For calculating maternal BMI, we used following formula:

BMI = Weight (in kilograms) / Height (in meters)^2^

1. In each DHS dataset, the weight of the respondent was presented in kilograms, while the height was presented in centimeters.
2. At first, we converted height in cemtimeters into meter.
3. We then put value of weight and height into the BMI formula, and thus calculated the BMI of each mother.
4. The calculated BMI was then coded numerically. A code “0” was assigned to all mothers having BMI ranged between 18.5 to 24.9 kg/m^2^, while code “1” and “2” was assigned to all underweight (<18.5 kg/m^2^) and overweight/obese (>25.0 kg/m^2^), respectively.

**S-3.2: Maternal height classification**

1. In each DHS dataset, the height is a continuous variable, which was then converted into a categorical variable by creating a new variable “MaternalHeight”
2. A cut-off value of 145 cm was used for classifying maternal height. Any mother height of 145 cm or more were classified as normal mother, and were coded “0”, while mother who failed to attain height of 145 cm were classified to be in the short stature category, coded “10”.
3. Thus, a code “0” was assigned to all Normal height mother, while code “10” designate “Short stature”.

**S-3.3: Identification of maternal malnutrition**

1. In this research, the maternal nutritional status was broadly classified into three classes: Normal, Standalone forms of malnutrition, and Coexisting forms of malnutrition.
2. Before maternal nutritional status classification, we performed computational analysis by adding the coding of two variables: MaternalBMI and MaternalHeight.
3. After computational analysis, we received following outcomes: 0,1,2,10,11,12.
4. Further coding was performed, where code 1 to 10 was coded as “1”, and those having code 11 and 12 were coded “2”. Individuals having code “0” were coded “0”.
5. Maternal nutritional status was labelled Normal against code “0”, Standalone forms of malnutrition against code “1”, and CFM against code “2”.

**Supplementary file 4**

**S-4: Unadjusted odds for assessing the determinants of various forms of coexisting forms of malnutrition.**

| Variable | Categories | Coexistence of underweight with wasting^∞^ | | Coexistence of underweight with stunting^∞^ | | Coexistence of underweight with wasting and stunting both^∞^ | | Coexistence of stunting with overweight/obesity^¥^ | |
| --- | --- | --- | --- | --- | --- | --- | --- | --- | --- |
|  |  | **Unadjusted Odds**  **(95% CI)^1^** | **p-value** | **Unadjusted Odds**  **(95% CI)^2^** | **p-value** | **Unadjusted Odds**  **(95% CI)^3^** | **p-value** | **Unadjusted Odds**  **(95% CI)^4^** | **p-value** |
| Year | **2012-2013** | Ref | 0.059 | Ref | 0.248 | Ref | 0.808 | Ref | <0.001 |
|  | **2017-2018** | 1.71 (0.97 to 3.01) |  | 1.34 (0.81 to 2.21) |  | 1.07 (0.61 to 1.87) |  | 0.28 (0.21 to 0.40) * |  |
| Age | **0-11 mo** | Ref | 0.036 | Ref | <0.001 | Ref | 0.004 | Ref | <0.001 |
|  | **12-23 mo** | 3.65 (1.29 to 10.31) * |  | 7.53 (2.85 to 19.90) * |  | 5.60 (2.03 to 15.40) * |  | 0.14 (0.07 to 0.26) * |  |
|  | **24-35 mo** | 2.63 (1.09 to 6.34) * |  | 6.66 (2.98 to 14.90) * |  | 1.94 (0.79 to 4.71) |  | 0.29 (0.18 to 0.47) * |  |
|  | **36-47 mo** | 1.78 (0.74 to 4.25) |  | 6.00 (2.77 to 12.97) * |  | 1.50 (0.62 to 3.58) |  | 0.26 (0.16 to 0.42) * |  |
|  | **48-59 mo** | 1.23 (0.56 to 2.64) |  | 3.07 (1.57 to 5.99) * |  | 1.18 (0.55 to 2.52) |  | 0.32 (0.19 to 0.53) * |  |
| Sex | **Male** | Ref | 0.514 | Ref | 0.680 | Ref | 0.742 | Ref | 0.222 |
|  | **Female** | 1.20 (0.68 to 2.11) |  | 1.11 (0.67 to 1.82) |  | 0.91 (0.52 to 1.58) |  | 1.21 (0.89 to 1.64) |  |
| Presence of illnesses | **No** | Ref | 0.159 | Ref | 0.252 | Ref | 0.577 | Ref | 0.041 |
|  | **Yes** | 1.50 (0.85 to 2.62) |  | 1.34 (0.81 to 2.19) |  | 1.17 (0.67 to 2.03) |  | 0.72 (0.53 to 0.98) * |  |
| Birth size | **Average** | Ref | 0.304 | Ref | 0.480 | Ref | 0.633 | Ref | 0.994 |
|  | **Large** | 0.47 (0.12 to 1.75) |  | 0.88 (0.31 to 2.54) |  | 0.53 (0.14 to 1.90) |  | 1.01 (0.57 to 1.76) |  |
|  | **Small** | 0.66 (0.34 to 1.28) |  | 0.69 (0.39 to 1.23) |  | 0.91 (0.48 to 1.71) |  | 0.97 (0.63 to 1.49) |  |
| Maternal age | **< 20 years** | Ref | 0.472 | Ref | 0.463 | Ref | 0.871 | Ref | 0.040 |
|  | **20 to 34 years** | 2.37 (0.611 to 9.17) |  | 2.11 (0.71 to 6.26) |  | 1.30 (0.39 to 4.33) |  | 0.31 (0.12 to 0.74) * |  |
|  | **35 year or more** | 2.13 (0.50 to 9.03) |  | 2.06 (0.63 to 6.64) |  | 1.15 (0.31 to 4.21) |  | 0.36 (0.14 to 0.92) * |  |
| Maternal education | **No education** | Ref | 0.420 | Ref | 0.360 | Ref | 0.061 | Ref | 0.185 |
|  | **Primary** | 0.61 (0.28 to 1.29) |  | 0.61 (0.32 to 1.17) |  | 0.43 (0.20 to 0.91) * |  | 0.63 (0.38 to 1.05) |  |
|  | **Secondary or higher** | 0.98 (0.49 to 1.93) |  | 0.93 (0.51 to 1.71) |  | 0.59 (0.30 to 1.18) |  | 0.91 (0.64 to 1.29) |  |
| Maternal working status | **No** | Ref | 0.028 | Ref | 0.139 | Ref | 0.281 | Ref | 0.588 |
|  | **Yes** | 0.45 (0.22 to 0.91) |  | 0.63 (0.35 to 1.13) |  | 0.69 (0.36 to 1.33) |  | 0.88 (0.56 to 1.38) |  |
| Maternal empowerment for healthcare | **No** | Ref | 0.622 | Ref | 0.884 | Ref | 0.191 | Ref | <0.001 |
|  | **Yes** | 1.16 (0.64 to 2.11) |  | 1.04 (0.61to 1.76) |  | 0.68 (0.38 to 1.22) |  | 0.52 (0.38 to 0.72) * |  |
| Maternal BMI ^α^ | **Normal** | Ref | 0.071 | Ref | 0.442 | Ref | 0.748 | Ref | 0.007 |
|  | **Underweight** | 0.39 (0.17 to 0.91) * |  | 0.82 (0.42 to 1.60) |  | 0.96 (0.46 to 2.01) |  | 0.88 (0.46 to 1.68) |  |
|  | **Overweight/obese** | 1.08 (0.56 to 2.09) |  | 1.32 (0.73 to 2.39) |  | 1.25 (0.65 to 2.42) |  | 1.61 (1.16 to 2.21) * |  |
| Maternal weight (cont.) ^α^ |  | 1.02 (0.99 to 1.04) | 0.196 | 1.01 (0.98 to 1.03) | 0.338 | 1.00 (0.98 to 1.03) | 0.744 | 1.01 (0.99 to 1.02) | 0.079 |
| Maternal height (cont.) |  | 0.99 (0.96 to 1.04) | 0.932 | 0.99 (0.95 to 1.03) | 0.675 | 0.99 (0.96 to 1.03) | 0.847 | 0.99 (0.96 to 1.02) | 0.806 |
| Maternal height | **Normal/tall stature** | Ref | 0.813 | Ref | 0.479 | Ref | 0.544 | Ref | 0.565 |
|  | **Short stature** | 0.88 (0.32 to 2.39) |  | 0.72 (0.30 to 1.73) |  | 0.73 (0.26 to 1.98) |  | 0.81 (0.39 to 1.67) |  |
| Total children delivered^α^ |  | 1.06 (0.93 to 1.19) | 0.365 | 1.07 (0.95 to 1.19) | 0.231 | 1.05 (0.93 to 1.18) | 0.441 | 1.06 (0.99 to 1.12) | 0.074 |
| Birth in last year | **No** | Ref | 0.348 | Ref | 0.009 | Ref | 0.330 | Ref | <0.001 |
|  | **Yes** | 0.76 (0.43 to 1.34) |  | 0.51 (0.31 to 0.84) * |  | 0.76 (0.43 to 1.32) |  | 1.69 (1.24 to 2.30) * |  |
| Delivery by C-section | **No** | Ref | 0.082 | Ref | 0.121 | Ref | 0.123 | Ref | 0.710 |
|  | **Yes** | 0.48 (0.22 to 1.08) |  | 0.58 (0.30 to 1.12) |  | 0.53 (0.25 to 1.16) |  | 1.08 (0.70 to 1.68) |  |
| Place of birth | **Home** | Ref | 0.887 | Ref | 0.602 | Ref | 0.730 | Ref | 0.255 |
|  | **Hospital** | 0.96 (0.55 to 1.68) |  | 1.14 (0.69 to 1.87) |  | 0.91 (0.52 to 1.57) |  | 0.83 (0.61 to 1.13) |  |
| Socioeconomic status | **Poorest** | Ref | 0.480 | Ref | 0.359 | Ref | 0.302 | Ref | 0.242 |
|  | **Poorer** | 1.30 (0.61 to 2.77) |  | 0.69 (0.35 to 1.36) |  | 0.87 (0.42 to 1.82) |  | 0.83 (0.54 to 1.28) |  |
|  | **Middle** | 1.15 (0.50 to 2.68) |  | 0.81 (0.38 to 1.69) |  | 0.65 (0.28 to 1.49) |  | 0.65 (0.40 to 1.06) |  |
|  | **Richer** | 1.91 (0.71 to 5.13) |  | 1.61 (0.65 to 3.93) |  | 1.17 (0.44 to 3.10) |  | 0.75 (0.45 to 1.25) |  |
|  | **Richest** | 0.71 (0.27 to 1.85) |  | 0.76 (0.34 to 1.70) |  | 0.39 (0.15 to 1.03) |  | 1.11 (0.71 to 1.76) |  |
| Family size |  | 1.05 (0.98 to 1.11) | 0.113 | 1.03 (0.97 to 1.09) | 0.243 | 1.07 (1.00 to 1.14) * | 0.032 | 1.00 (0.97 to 1.02) | 0.922 |
| Type of place of residence | **Rural** | Ref | 0.190 | Ref | 0.913 | Ref | 0.444 | Ref | 0.417 |
|  | **Urban** | 1.46 (0.82 to 2.59) |  | 1.03 (0.61 to 1.72) |  | 1.25 (0.71 to 2.19) |  | 1.13 (0.83 to 1.54) |  |

∞ = The reference category for assessing the determinants of the coexistence of underweight with wasting, coexistence of underweight with stunting, and coexistence of underweight with wasting and stunting both was stunting.

¥ = The reference category for assessing the determinants of the coexistence of stunting with overweight/obesity was stunting.
